# Development, Acceptability, and Initial Implementation of an Interactive Text-Messaging Program for Fathers with Low Income

**DOI:** 10.1007/s10995-024-03983-7

**Published:** 2024-08-09

**Authors:** Joyce Y. Lee, Shawna J. Lee, Amy Xu, Hannah Steinke, Christina Weiland

**Affiliations:** 1 The Ohio State University College of Social Work, 1947 North College Road, Columbus, OH 43210 USA; 2 University of Michigan, Ann Arbor, USA

**Keywords:** Father Engagement, Maternal and Child Health, Home Visiting, Text4Dad, Implementation, Technology and Families

## Abstract

**Objectives:**

This study describes the development, acceptability, and implementation of an interactive text messaging program to engage fathers enrolled in home visitation programs.

**Methods:**

We used an iterative development approach that integrated rapid testing of intervention content with acceptability feedback from program participants to examine the processes of implementation. In Study 1, we describe the rapid testing framework and present data from 171 men who provided feedback on Text4Dad content via three online surveys. In Study 2, a case study, we use administrative data from 108 fathers with whom we pilot-tested Text4Dad in three community-based home visiting programs, with the program implemented by fatherhood program community health workers (F-CHWs). Content analysis of exchanges between F-CHWs and fathers describes the specific use of Text4Dad.

**Results:**

Across all three online surveys, fathers reported positive reviews of the Text4Dad content. The F-CHWs used Text4Dad mainly to push out information, especially that related to home visit scheduling and local events, instead of engaging in bidirectional interactions with fathers.

**Conclusions for Practice:**

We conclude with a set of recommendations for social service and maternal and child health providers regarding the feasibility of implementing text messaging to support home visiting in community-based settings.

## Significance

What is Already Known on this Subject?

What this Study adds?

In leveraging mobile technology for behavioral change, there has been a proliferation of text messaging parenting interventions. Innovative texting and technology-focused programs for fathers have been developed and implemented, with a focus on improving fathering quality, father-child interactions, program fidelity, and measures relevant to the duration, timing, and context of programs. That said, the effectiveness of these programs has been challenging to ascertain and compare, given and unique programmatic aspects of each intervention and lack of robust program evaluation designs.

Most father-inclusive text messaging interventions are stand-alone programs. Importantly, Text4Dad is one of the first programs to serve as an add-on to an existing community-based home visitation model, with the goal of engaging fathers who are from low-income contexts and receiving in-home visits. This initial implementation study shows the promise of a low-cost and mentor-based text messaging program that is appealing and useful for fathers and male CHWs serving them.

## Introduction

Phone ownership and mobile technology usage are ubiquitous. For example, the Pew Research Center (Perrin, 2021) reported that the larger majority of adults (83%)—including those with low income of less than $30,000 per year—own a cell phone, and 73% of them use text messaging on a regular basis. Young adults are the most frequent users of text messaging, with 18–24 year olds sending or receiving an average of 109.5 messages per day and 25–34 year olds 41.8 messages per day, compared to 9.8 messages amongst 55–64 year old adults (Ashford et al., 2018; Smith, 2011). Recognizing that technology can be used to support behavioral change, there has been a proliferation of text messaging interventions across domains of health, mental health, and parenting (Badawy & Kuhns, 2017; Riley et al., 2011).

Recognizing the importance of using technology to reach new parents, a number of innovative texting and technology-focused programs for fathers have been developed (Balu et al., 2018, 2021; Hayward et al., 2021; Jabaley et al., 2011; S. J. Lee & Walsh, 2015; Lewin-Bizan et al., 2020; Marcell et al., 2021; May & Fletcher, 2019; Self-Brown et al., 2018), many of which were developed as stand-alone programs and without an explicit mentoring or interactive component. In this study, we examine Text4Dad—a mentor-based, interactive text messaging program—implemented in conjunction with home visiting services delivered to fathers in community-based settings. Text4Dad was developed specifically to support interactions with fathers and mentors in-between home visits, and thus was intended to supplement in-person home visiting services (Wulfsohn et al., 2021). We describe two components of developing and testing Text4Dad. In Study 1, we used an innovative framework called rapid prototyping (Balu et al., 2018) to test components of the Text4Dad messaging using online surveys. In Study 2, we present a case study in which we pilot-tested Text4Dad with fathers who were participating in a community-based home visiting program.

## Background of the Text4Dad Program

Text4Dad was adapted from the NurturePA program, which was developed to provide peer support mentorship to postpartum mothers using personalized text messaging (Martin et al., 2020; Weiland et al., 2023). Female volunteers who were mothers themselves were trained to provide emotional support, answer questions, encourage activities promoting maternal wellbeing and healthy infant development, and direct mothers to relevant local resources (Martin et al., 2020; Weiland et al., 2023). Similar to Text4baby (Evans et al., 2012), the NurturePA platform included some messages that were pre-formulated and vetted by child development experts to create a curated repository of evidence-based text messages. However, unlike Text4baby, the communication between mothers and mentors was two-way and mentors also were not constrained to the pre-formulated messages (i.e., they could tailor them and engage in more naturalistic conversation beyond the pre-formulated messages).

Several key findings emerged in a NurturePA implementation study with 162 mom-mentor dyads (Martin et al., 2018). Half of the enrolled mothers were first-time mothers, with 77% of them communicating at least one problem during their involvement in NurturePA. In instances where a problem was identified, the mentors provided advice (35%), emotional support (39%), website information (15%), and referrals (13%) among other types of support. Furthermore, a recent pre-registered pilot randomized control trial of NurturePA (Weiland et al., 2023) showed that most enrolled mothers stayed engaged for the full 18-month project period and that mother-mentor dyads primarily discussed via text maternal and child health topics (e.g., child socioemotional and language development, child sleep, maternal wellbeing). There were no impacts on targeted maternal or child outcomes, though some impacts were meaningful and large (> 0.2 SDs) in magnitude.

Researchers have begun to consider how to use similar approaches to meet fathers’ parenting needs (Balu et al., 2018, 2021; Hayward et al., 2021; Lee & Walsh, 2015; Lewin-Bizan et al., 2020; Marcell et al., 2021; May & Fletcher, 2019; Self-Brown et al., 2018). Existing research on technology-focused father interventions is largely focused on improving fathering quality and increasing father-children interaction time, and although fathering measures vary from program to program, such programs have been received positively by fathers, families, caseworkers, or all of the above (Marcell et al., 2021; May & Fletcher, 2019; Fletcher et al., 2024, Self-Brown et al., 2018; Balu et al., 2021; Lewin-Bizan et al., 2020; S. J. Lee & Walsh, 2015; Hayward et al., 2021).

Prior research has also focused on questions related to intervention implementation, program fidelity, and measures that are relevant to the duration, timing, and context of the specific program (Marcell et al., 2021; May & Fletcher, 2019; Fletcher et al., 2024, Self-Brown et al., 2018; Balu et al., 2021; Lewin-Bizan et al., 2020; S. J. Lee & Walsh, 2015; Hayward et al., 2021). However, the existing literature base also shows that interventions which are both father-focused and technology-based are few, and their effectiveness can be difficult to compare given unique programmatic aspects of each intervention and lack of robust program evaluation designs (Marcell et al., 2021; May & Fletcher, 2019; Fletcher et al., 2024, Self-Brown et al., 2018; Balu et al., 2021; Lewin-Bizan et al., 2020; J. Y. Lee et al., 2018; S. J. Lee & Walsh, 2015; Hayward et al., 2021).

A number of U.S.-based texting programs for fathers are being developed and tested in the field. For example, Fathers and Babies (FAB) is an intervention with both texting and in-person delivery methods to support fathers’ mental health during the transition to parenthood (Tandon et al., 2021). FAB begins with an initial session in person or by phone, and then based on fathers’ preference, subsequent sessions are in person, by text, or a mix of both. The program is integrated into an existing home visiting program and thus father participants in FAB are partnered with mothers who receive home visits. Another relevant program, text4FATHER, is designed to focus on caring for a new baby, and one study to date describes the feasibility, acceptability, and preliminary efficacy trial from mid-pregnancy through two months postnatal age (Marcell et al., 2021). Relatedly, DadTime app (Balu et al., 2018, 2021) aimed at encouraging fathers’ attendance in the Just Beginning program, an in-person parenting program for fathers of young children (Harknett et al., 2017; Israel et al., 2017). DadTime content helped fathers to anticipate and address barriers in getting to the Just Beginning in-person sessions (Balu et al., 2021).

Overall, preliminary results of the aforementioned interventions have been small in scope but promising. For example, fathers participating in FAB reported reductions in depressive symptoms and increases in high levels of emotional and instrumental support, and DadTime app content helped fathers anticipate and address barriers to attending in-person sessions (Balu et al., 2018, 2021; Tandon et al., 2021). From semi-structured interview data, fathers from SMS4dads identified that text message interventions were helpful, reduced isolation, and supported father-infant and father-partner relationships (May & Fletcher, 2019). However, additional research, including more robust evaluation designs with comparison groups, is needed to capture greater evidence of effectiveness (Tandon et al., 2021). In one case in which a randomized control trial involving the DadTime app was conducted, the intervention was not effective in increasing father attendance (Balu et al., 2021).

## The Text4Dad Program: Approach, Content, Population, and Implementation Context

To meet fathers’ early parenting needs, as well as to support and engage fathers enrolled in-person home visiting programs, we created the Text4Dad program. The NurturePA team developed Text4Dad’s text messaging software platform. While Text4Dad and NurturePA shared a similar platform, the content, approach, and populations of these programs were different. First, all Text4Dad content was developed via prior research (J. Y. Lee & S. J. Lee, 2023) and then tested to be appropriate for fathers (see Study 1 below). As such, Text4Dad did not use any of NurturePA’s content, which was designed with mothers in mind. Second, using an approach more similar to that of DadTime, Text4Dad was designed as an add-on to an *in-person* fatherhood home visiting program, with fatherhood community workers making regular home visits to meet with and support enrolled fathers. In contrast, NurturePA female peer mentors never had in-person interactions with their new mother mentees. Finally, Text4Dad primarily focused on fathers with low income in the community, whereas NurturePA focused on new mothers from a range of sociodemographic backgrounds (Weiland et al., 2023).

Text4Dad was implemented within the context of and in collaboration with three Healthy Start programs in the community (J. Y. Lee & S. J. Lee, 2023). Healthy Start is a national program funded by the U.S. Department of Health and Human Services and aimed at improving maternal and child health outcomes, especially in local communities experiencing high levels of adverse outcomes (e.g., infant mortality, preterm births, maternal illness) (U.S. Department of Health & Human Services, 2023). Though services were primarily focused on mothers, in each of the three participating Healthy Start programs, there was one Fatherhood Community Health Worker (F-CHW) employed to provide home visitation and parent education to fathers who were the partners of mothers enrolled in the program. Thus, Text4Dad was developed so that the three F-CHWs could communicate via text with the fathers on their caseloads in between home visits.

Text4Dad messages were designed to focus on content delivered to fathers in the home visitation component of the program, reinforce information delivered in the visit, and potentially serve as “booster shots.” Text4Dad messaging content addressed six areas previously identified by fathers as being important (e.g., see Study 1 below): (1) infant development, including physical, cognitive, and socioemotional development; (2) father-infant attachment and bonding; (3) fathers’ infant caregiving; (4) father-infant play, with developmentally appropriate activities; (5) father-mother coparenting; and (6) fathers’ self-care. In addition, check-in messages meant to encourage two-way interactions were developed that asked fathers how they and their families were doing, whether the messages and online resources sent were helpful, and if they needed any additional support or information from the F-CHWs. Finally, Text4Dad included a curated list of web links of existing father-friendly evidence-based resources that F-CHWs could send as needed.

## The Current Study

The current study describes the development and implementation of Text4Dad. In Study 1, we report on the acceptability and usability of Text4Dad, using procedures adapted from a rapid prototyping approach (Balu et al., 2018; IDEO.org, 2015). This approach was used to examine the *content* of the Text4Dad messages, with survey data collected from three short online studies. We used collected data to validate the content of the messages, and when necessary, refine or modify the messages in accordance with feedback from survey respondents. In Study 2, a case study, we describe the *implementation* of Text4Dad in the context of a community-based home visiting program to explore how F-CHWs use the program to engage fathers receiving home visits. Consistent with prior NurturePA studies (Martin et al., 2018; Weiland et al., 2023), we examined usage data from the Text4Dad text message platform and conducted content analysis of exchanges between the F-CHWs and fathers (e.g., how long F-CHWs and fathers used the program, how often they exchanged messages, who more commonly initiated interactions, and the nature of the interactions). We present data on the topics of text exchanges and the online resources provided to fathers. A separate qualitative analysis of F-CHWs and fathers on their experiences using Text4Dad is described elsewhere (J. Y. Lee & S. J. Lee, 2023). For both Study 1 and Study 2, the Institutional Review Board (IRB) at University of Michigan provided approval, and we report research elements that align with STrengthening the Reporting of OBservational studies in Epidemiology (STROBE) (https://www.strobe-statement.org).

## Study 1: Acceptability and Usability of Text4Dad Content

### Methods

For Study 1, we used a rapid prototyping approach described in Balu et al. (2018), which is a human-centered design process in which multiple iterations of a product are created based on user feedback. Feedback from rapid prototyping sessions was used to refine the content and format. In our case, we used several short surveys with fathers to obtain feedback on the content of Text4Dad messages. Specifically, three online surveys were deployed between August 2017 and March 2019, using participants from Amazon Mechanical Turk (MTurk)—a crowdsourcing platform that is widely used in behavioral research (Casler et al., 2013; Peer et al., 2017). MTurk allows workers to do tasks (e.g., complete surveys) for pay, with all of the work completed online. Thus, Study 1 consists of a convenience sample of fathers recruited from MTurk who completed brief online surveys for pay. Fathers received between $1 and $3 for completing the surveys, which on average took 21 min.

The goal of the first online survey was to examine fathers’ preferences for standard English text messages versus text language messages (e.g., “How R U 2 day?”). Specifically, fathers were randomized into either a standard English or a text language message group. The purpose of the second online survey was to examine the acceptability and usability of a series of Text4Dad messages divided into a number of parenting topics (e.g., caregiving, self-care) by infants’ developmental stages (i.e., 0–2, 2.1-4, 4.1-9, 9.1–15 months). The purpose of the third online survey was to examine the acceptability and usability of Text4Dad’s messages specifically with a sample of fathers from low-income contexts, as fathers from the first two surveys were largely middle income and did not reflect the Healthy Start population.

### Participants and Procedures

For all three MTurk surveys, eligible participants had to be male, between the ages of 18 and 40 years, fluent in English, have a U.S.-based IP address, and have a positive MTurk work history (i.e., to ensure data quality and prevent potential bots) (Peer et al., 2014). The first MTurk survey involved *n* = 50 men expecting a baby or who had a child under three years old. Sample characteristics can be found in Table 1. Approximately half of the fathers were randomly assigned to the standard English message group (*n* = 24) and the other half to the text language message group (*n* = 26). As shown in Table 1, balance checks comparing the two randomized groups on a number of sociodemographic covariates revealed statistically significant differences between the two groups on two race categories (e.g., White, Other). That said, the overall balance test was not statistically significant, *F*(14, 28) = 1.09, *p* = .406, suggesting that the sample was generally balanced despite imbalances on a few sociodemographic characteristics.

**Table 1:** Sample characteristics and Text4Dad message ratings of online surveys 1–3

	Online Survey 1 (*N* = 50)			Online Survey 2 (*N* = 99)	Online Survey 3 (*N* = 16)
	Text language (*n* = 26)	Standard English (*n* = 24)		--	--
Variable	*M* (*SD*) or *n* (%)	*M* (*SD*) or *n* (%)	*p*-value	*M* (*SD*) or *n* (%)	*M* (*SD*) or *n* (%)
*Background Characteristics*					
Father’s age	32.44 (4.47)	31.83 (4.41)	0.635	33.43 (52.24)	32.56 (4.77)
Father’s race/ethnicity:					
White	13 (50.00%)	19 (79.17%)	0.032*	78 (78.79%)	9 (56.25%)
Black	2 (7.69%)	3 (12.50%)	0.571	3 (3.03%)	1 (6.25%)
Hispanic/Latinx	1 (3.85%)	0 (0.00%)	0.332	9 (9.09%)	1 (6.25%)
Other	10 (38.46%)	2 (8.33%)	0.013*	9 (0.09%)	5 (31.25%)
Father’s education:					
Some HS or HSD/GED	2 (7.69%)	3 (12.50)	0.571	8 (8.16%)	5 (31.25%)
Some college	6 (23.08%)	8 (33.33%)	0.420	32 (32.65%)	5 (31.25%)
Bachelor’s degree	15 (57.69%)	12 (50.00%)	0.586	46 (46.94%)	3 (18.75%)
Graduate degree	3 (11.54%)	1 (4.17%)	0.337	12 (12.24%)	3 (18.75%)
Father is employed	26 (100%)	20 (90.91%)	0.116	94 (95.92%)	15 (93.75%)
Father’s annual income			0.722		
Less than $20,000	3 (13.04%)	2 (8.70%)		2 (2.17%)	5 (31.25%)
$20,001-$30,000	3 (13.04%)	3 (13.04%)		9 (9.78%)	8 (50.00%)
$31,001-$40,000	2 (8.70%)	3 (13.04%)		10 (10.87%)	3 (18.75%)
$40,001-$50,000	2 (8.70%)	5 (21.74%)		13 (14.13%)	--
More than $50,000	13 (56.52%)	10 (43.48%)		58 (63.04%)	--
Father is married	18 (69.23%)	16 (66.67%)	0.846	66 (66.67%)	6 (37.50%)
Father’s number of children	1.42 (0.76)	1.48 (0.73)	0.797	1.57 (0.83)	1.13 (0.34)
*Text4Dad Message Ratings*					
Likability of the text messages (range: 1–5)^†^	2.88 (1.73)	4.46 (0.88)	--	--	--
Overall acceptability and usability of text content (range: 1–7)	5.99 (1.16)	6.00 (0.72)	0.967	5.94 (0.99)	5.86 (1.36)

Note. HS = High School; HSD = High School Diploma; GED = General Education Development. Analysis of variance was used for continuous variables and a chi-square test was used for categorical variables. ^†^Fathers assigned to each group (standard English vs. text langauge) were asked how much they liked the text messages. *p < .05

The second MTurk survey involved *n* = 99 men participating under similar eligibility criteria as those of the first study, although participants had to have a child between the ages of 0 and 15 months. The third MTurk survey involved *n* = 16 fathers with the same eligibility criteria as those of the second MTurk survey, but the fathers needed to be from low-income contexts (e.g., annual household incomes less than or equal to $40,000). Table 1 provides sample characteristics of all three online surveys. Table 2 provides the distribution of fathers across infant age categories pertaining to the second and third surveys.

**Table 2:** Assessment of how much fathers liked the Text4Dad messages and how much they perceived the messages to be father-friendly by content topic and child age in months

Content topic*	Online Survey 2 (*N* = 99)				Online Survey 3 (*N* = 16)			
	0–2 m (*n* = 7)	2.1–4 m (*n* = 22)	4.1–9 m (*n* = 36)	9.1–15 m (*n* = 34)	0–2 m (*n* = 1)	2.1–4 m (*n* = 8)	4.1–9 m (*n* = 4)	9.1–15 m (*n* = 3)
How much fathers liked particular Text4Dad messages, *M* (*SD*) (range:1–5)								
Infant development	4.29 (1.50)	4.41 (0.73)	4.36 (0.83)	4.38 (0.89)	5.00 (--)	4.35 (1.39)	3.75 (1.50)	4.00 (1.00)
Attachment	4.14 (1.46)	4.45 (0.74)	4.56 (0.73)	4.18 (1.09)	5.00 (--)	4.38 (0.74)	4.25 (0.96)	4.33 (0.58)
Caregiving	4.43 (1.51)	4.05 (0.95)	4.44 (0.61)	4.15 (1.02)	5.00 (--)	4.00 (1.60)	4.25 (0.05)	3.67 (1.53)
Play	4.00 (1.41)	4.45 (0.74)	4.56 (0.56)	4.35 (0.73)	5.00 (--)	4.50 (0.76)	4.25 (0.96)	4.67 (0.58)
Coparenting	4.57 (0.79)	3.68 (1.21)	3.94 (1.04)	4.00 (0.85)	5.00 (--)	4.13 (0.83)	3.00 (1.83)	4.67 (0.58)
Self-care	4.12 (1.46)	4.00 (1.02)	4.39 (0.77)	4.29 (0.94)	5.00 (--)	4.38 (0.92)	3.75 (1.26)	3.67 (0.58)
How much fathers perceived Text4Dad Messages to be father-friendly, *M* (*SD*) (range: 1–5)								
Infant development	4.14 (1.46)	4.32 (0.72)	4.36 (0.80)	4.21 (0.81)	5.00 (--)	4.35 (1.39)	3.50 (1.29)	4.33 (0.58)
Attachment	3.86 (1.68)	4.36 (0.79)	4.53 (0.65)	4.15 (0.93)	5.00 (--)	4.50 (0.76)	4.25 (0.50)	4.00 (1.00)
Caregiving	4.29 (1.50)	4.23 (0.81)	4.39 (0.64)	4.09 (0.87)	4.00 (--)	4.00 (1.60)	3.50 (1.00)	3.00 (1.00)
Play	4.14 (1.46)	4.60 (0.50)	4.42 (0.60)	4.26 (0.79)	5.00 (--)	4.50 (0.76)	4.00 (0.82)	4.33 (0.58)
Coparenting	4.29 (1.11)	4.05 (1.25)	3.83 (1.10)	4.24 (0.74)	5.00 (--)	4.50 (0.76)	3.00 (1.83)	4.67 (0.58)
Self-care	4.00 (1.41)	4.23 (0.97)	4.44 (0.61)	4.26 (0.86)	5.00 (--)	4.38 (0.92)	4.00 (0.82)	3.67 (0.58)

Note. *Each content topic entailed fathers reading three example text messages tailored to the child’s specific age category

We had concerns about bot issues with MTurk and thus, in accordance with expert recommendations (e.g., Buchanan & Scofield, 2018; Hunt & Scheetz, 2019; Xu et al., 2022), we put in place a number of prevention strategies to safeguard against fraud stemming from bots. These prevention strategies included using high levels of qualifications (i.e., Human Intelligence Tests (HITs) approved > 1000, 99% approval percentage and above, locations in the U.S. only), employing CAPTCHAs to make sure the survey was completed by humans, incorporating attention checks at various parts of the survey, manually reviewing HIT submissions regularly as part of quality checks, and analyzing data for patterns (e.g., completing survey within a very short amount of time, text responses being identical across participants) among others.

### Measures

In the first online survey with randomly assigned groups, fathers were asked about how much they liked the messages assigned to them (i.e., standard English vs. text language): “How much do you like these messages?” using a 5-point scale (1 = *dislike a lot*, 5 = *like a lot*). All three surveys used overall acceptability and usability measures with eight items (e.g., “Overall, I am satisfied with the text messages”). Fathers rated their agreement with the overall acceptability and usability of the text messages on a 7-point scale (1 = *strongly disagree*, 7 = *strongly agree*), with all three surveys showing good reliability of the measure (survey 1: α = 0.91; survey 2: α = 0.90; survey 3: α = 0.95). For the second and third online surveys, fathers were presented with developmentally appropriate (i.e., tailored to their infants’ age categories) text messages that were presented in six different content areas (i.e., infant development, attachment and bonding, caregiving, play, coparenting, and self-care). For each content area, fathers were presented with three example text messages and were asked to rate whether they liked the messages (i.e., “How much do you like these messages?”) on a 5-point scale (1 = *dislike a lot* to 5 = *like a lot*), as well as whether the messages were father-friendly (i.e., “Are these messages father friendly?”) on a 5-point scale (1 = *strongly disagree* to 5 = *strongly agree*). Short qualitative responses were also collected in all three surveys (e.g., “Please give at least one specific reason why you think these messages are or are not father friendly”).

### Data Analysis

For all three online surveys, descriptive statistics were calculated for father-reported acceptability and usability of the Text4Dad messages, with bivariate analyses (i.e., chi-square tests and analysis of variance) conducted to compare differences between the two randomized groups. For the first survey only, descriptives for fathers’ reports of their preferences for standard English or text language-based messages were calculated. For the second and third surveys only, descriptives for fathers’ reports of how much they liked the messages and the father-friendly nature of the messages were calculated by content area and infants’ ages. Across all three surveys, common themes (e.g., additional topics to include, ways to improve content) from qualitative responses were identified and synthesized.

### Results

Overall, participants from the first survey reported high acceptability and usability of the Text4Dad messages (*M* = 5.95, *SD* = 1.16), with no significant differences between the standard English and text language groups (*p* = .967) (for details, see Table 1). Of note, fathers randomized to the text language message group expressed a general dislike for text language messages and a preference for standard English messages, whereas those randomized to the standard English language message group reported a general like for standard English messages compared to text language messages (Table 1). In their written responses, fathers noted their reasons for preferring standard English messages: “Some people probably wouldn’t understand it (text language messages),” and “Use of proper grammar and standard English gives the text more credibility to me.” Fathers from the first survey also suggested other important text message topics to include (e.g., caregiving, self-care, supporting mothers). Qualitative responses supported the high levels of father-reported acceptability and usability of the messages (e.g., “I love all the resources and information”). Additionally, fathers noted the importance of being personal in tone.

In the second survey, participants once more reported high acceptability and usability of the Text4Dad messages (*M* = 5.94, *SD* = 0.99). How much the fathers liked particular text messages (i.e., of different content areas) and their perceptions of father-friendliness of the text messages varied by children’s ages (Table 2). For example, fathers with 0–2 and 9.1–15 month-olds reported that the mother-father coparenting messages were most appealing to them, and those with 2.1-4 and 4.1-9 month-olds found the father-infant attachment messages most appealing. In their qualitative responses, fathers noted that the messages were encouraging, developmentally appropriate, and focused on practical things they could do.

In the third survey, fathers from low-income contexts also reported high acceptability and usability of the Text4Dad messages (*M* = 5.86, *SD* = 1.36). Similar to the second survey, how much fathers liked the messages and their perceptions of father-friendliness of the messages varied by children’s ages (Table 2). For instance, fathers with 2.1-4 and 9.1–15 month-olds found the father-infant play messages appealing, and fathers with 2.1-4 and 4.1-9 months-old perceived the self-care messages to be father-friendly to them. In their qualitative responses, fathers noted that the messages provided specific and useful suggestions they could put into practice. They also found the content of the messages generally encouraging and motivating to them.

## Study 2: A Case Study of Implementing Text4Dad

### Methods

#### Description of Text4Dad Sites and Program Implementation

Three of the six sites involved in the Healthy Start Engaged Father program multi-site evaluation participated in the Text4Dad pilot study. Participation required that one F-CHW at each site agree to use Text4Dad and participate in training and supervision by the University of Michigan research team. Hereafter, the three sites are referred to as Site 1 (small urban), Site 2 (large urban), and Site 3 (small urban). Of note, in order to host a Healthy Start site, the county must demonstrate adverse maternal and child health outcomes for, specifically, infant mortality rates at least 1.5 times the U.S. national average and/or high rates of preterm birth, low birth weight, and maternal illness and death among racially and ethnically underrepresented groups in the region. The sites were distributed geographically in one Midwestern rust belt state.

Site 1 is located in the center of the state. It is the third largest city in the state with a population of about 540,000. This city is about 21% Black and approximately 13% of families live in poverty. Overall, Site 1 is relatively advantaged compared to Sites 2 and 3. However, as noted, individuals and communities of color in these cities encounter significant health disparities and high poverty rates. Site 2 is located in the eastern part of the state, and is the most populated city in the state. Site 2 has suffered economically for decades due to declines in manufacturing and population. Site 2 is racially segregated, with about 75–80% of the city residents being Black or mixed race. About 34% of families live in poverty. Site 3 is located in the western part of the state, in a city with a population of approximately 260,000, with a population that is approximately 22% Black. About 14% of families live below the poverty line. In the public school system, there is a 65% high school graduation rate, which is notably lower than surrounding areas or the statewide average.

To implement Text4Dad, F-CHWs invited fathers from their home visitation caseloads to participate in the program. The first few months of the project focused on developing the Text4Dad F-CHW training manual and establishing procedures for recruitment and incentives for the participants at each program site. F-CHW training took place in the fall of 2018, which included how to access the Text4Dad software platform, add participants, select text messages, and send individual and group messages. Text4Dad was subsequently launched at Site 1 and Site 3 in October 2018. Given shortages in staffing, Site 2 did not begin implementing Text4Dad until late spring of 2019. Data collection ended in July 2019, and data were collected at varying intervals. Based on the site in which fathers were enrolled, data were collected for 288 days at Site 1, 48 days at Site 2, and 275 days at Site 3.

In addition to training, the Text4Dad research team provided administrative support for all sites, including sending weekly emails to the F-CHWs with sample messages and ideas for sending content to enrolled fathers. Weekly Text4Dad messages were meant to serve as add-ons or complementary resources alongside in-person home visits and community-based activities that varied in frequency by site. The research team also provided ongoing technical assistance through monthly learning community meetings amongst F-CHWs and additional booster sessions for how to use Text4Dad and its platform. Tailored technical assistance to each site was provided through scheduled phone calls and on an as-needed basis.

### Participants

Each F-CHW aimed at and recruited a minimum of 30 fathers to participate in Text4Dad, and thus a total of 108 fathers enrolled for Study 2 across all three sites (for details, see Table 3). The research team distributed $50 Visa gift cards to the F-CHWs once per month for approximately the first six months of program implementation to thank them for supporting the Text4Dad program. Furthermore, $25 Visa gift cards were made available to the F-CHWs to distribute to enrolled fathers for their participation in Text4Dad.

**Table 3:** Summary of descriptive and content analysis of Text4Dad exchanges between Fatherhood Community Health Workers and fathers

	Total (*N* = 108)	Site 1 (*n* = 32)	Site 2 (*n* = 45)	Site 3 (*n* = 31)
Number of days of using Text4Dad, from enrollment of first father to July 31, 2019 (last day data were collected for analysis)	611 days	288 days	48 days	275 days
Total # of exchanges* initiated by F-CHW	1,241	411	315	515
Average # of exchanges for each father	12.1	12.8	7	16.6
Total # of exchanges initiated by father	11 (6)	0 (0)	0 (0)	11 (6)
Primary exchange topic initiated by father	Scheduling, community events, and personal needs	N/A	N/A	Scheduling, community events, and personal needs
Primary exchange topic initiated by F-CHW:				
1. Father-infant attachment and bonding	67	69	--	1
2. Infant caregiving	77	24	1	52
3. Infant development	145	--	45	100
4. Father-infant play	17	--	--	17
5. Father-mother coparenting	53	53	--	--
6. Father’s self-care	170	54	90	26
7. Program functioning (e.g., enrollment, data collection, termination, etc.)	47	--	45	2
8. Check-in or scheduling home visits	133	1	--	132
9. Local or community-based events	538	210	134	194
10. Other	4	--	--	4
Total # of online resources offered	7	1 (i.e., paternal postpartum depression link)	2 (i.e., flyer for event, registration for focus group)	4 (i.e., bedtime routines, self-care and work/life balance, bonding with new baby, infant massage)

Note. F-CWH = Fatherhood Community Health Worker. *An “exchange” is defined as the initiation of an interaction by sending a text message within a given week and can consist of a single message or multiple messages; it is called an exchange because each message offers the possibility of an interactive exchange between an F-CHW and a father

### Measures

A number of measures were used in analyzing the text messaging exchanges that occurred between the F-CHWs and enrolled fathers and captured on the Text4Dad platform. These measures included *number of days of fathers using Text4Dad*, *total number of exchanges initiated by the F-CHW*, *total number of exchanges initiated by the father*, *number of online resources offered*, and *most common Text4Dad topics* among other measures (Table 3). Consistent with Martin et al. (2018), an “exchange” is defined as the initiation of an interaction by sending a text message within a given week and can consist of a single message (e.g., F-CHW sent a message and there was no response from father) or multiple messages (i.e., F-CHW sent a message and father responded or the F-CHW followed up with an additional prompt or question).

### Data Analysis

To conduct descriptive analysis of the F-CHW-father exchanges, we used administrative data available through the Text4Dad platform. Such data included the dates and times when F-CHWs and fathers sent messages, the content of the exchanges, and online resource links F-CHWs embedded into the sent Text4Dad messages. We calculated descriptive statistics for the duration, average number, and total number of exchanges, as well as the number of online resources offered by F-CHWs. Separately, content analysis of the F-CHW-father exchanges involved analyzing transcripts downloaded from the Text4Dad platform. We used the transcripts to code for the topics of exchange initiated by the fathers, types of resources F-CHWs sent out, and types of topics that were most common at each site. Three graduate-level students were trained on content coding for these fields and were blind to the identities of sites, F-CHWs, and enrolled fathers. Initially, the three graduate-level students independently coded content and then came together as a group to compare results. Where there was disagreement, the coders used extensive memo-taking, discussion, as well as consulting the lead researcher to reach consensus. The coding procedures were based on those developed by the NurturePA team (Martin et al., 2020), who trained our team in their data extraction and content coding process.

### Results

Table 3 provides a detailed summary of the descriptive and content analysis results. Sites 1 and 3 frequently employed Text4Dad and even shared a similar number of days in which fathers used Text4Dad. Site 2 had a substantially lower number of days in which fathers used Text4Dad compared to the two other sites, in part, because Site 2 started program implementation at a later date than the other two sites as noted above. However, Site 2 was able to enroll more fathers, in part because Site 2 is located in a larger urban area compared to the other two sites. Regarding the total number of exchanges initiated by the F-CHWs, Site 3 had the smallest number of enrolled fathers but had the largest number of F-CHW-initiated exchanges, at 16.6 exchanges per enrolled father. Furthermore, Site 3 was the only site in which exchanges were initiated by fathers (approximately 6 out of 31).

The results from the content analysis of the exchanges between the F-CHWs and fathers revealed that two out of three F-CHWs (i.e., from Sites 1 and 2) did not use Text4Dad to initiate sustained interactions with their fathers. Relatedly and as noted elsewhere, qualitative accounts of F-CHWs revealed that the F-CHWs largely did not use Text4Dad to provide mentoring and social support to enrolled fathers per se (J. Y. Lee & S. J. Lee, 2023). Rather, the F-CHWs employed the program to send parenting resources and reminders of upcoming home visit sessions or community-based fatherhood events. Interruptions and a late start to program implementation may have partly contributed to this pattern. For example, Site 1 had an interruption in programming during the study period with the F-CHW going on paternity leave, and Site 2 started its implementation almost 1.5 years later compared to the other sites given staff shortages. Nearly all of the exchanges for these F-CHWs consisted of one text message sent to a father per week, with no efforts to elicit responses from the fathers, no responses received, and thus no interactions taking place. For Site 3, when fathers initiated exchanges, exchange topics were around home visit schedules, community events, and personal needs. For example, fathers asked for confirmations of home visit times, clarification on community-based events, and material goods (e.g., dress shoes, housing voucher, bus token).

With regards to primary topics as initiated by F-CHWs, information on local or community-based events was most common across all sites (Table 3). Notably, the F-CHW from Site 3 used check-in messages, which included prompts that encouraged fathers to respond back, to a greater degree than did F-CHWs from Sites 1 and 2, leading to more interactive exchanges with fathers. Some variations in parenting topics emerged as well across sites. For example, the F-CHW at Site 1 commonly sent out messages pertaining to father-infant attachment, whereas the F-CHW at Site 2 commonly sent out messages related to fathers’ self-care practices. Relatedly, qualitative accounts of F-CHWs showed that F-CHWs had mixed responses to integrating Text4Dad topic contents to home visits, with the F-CHW at Site 3 having conversations with fathers during home visits about parenting topics introduced via Text4Dad, but the other two F-CHWs at Sites 1 and 2 noting that they felt that parenting-related conversations are best saved for in-person home visits (J. Y. Lee & S. J. Lee, 2023).

## Discussion

The current study described the development, acceptability, and initial implementation of Text4Dad. Innovative features and contributions of this study include testing Text4Dad as an add-on to an existing home visitation model, adapting rapid prototyping procedures (Balu et al., 2018; IDEO.org, 2015), and exploring how F-CHWs may engage fathers enrolled in home visits using a mobile technology-based program. By doing so, we have demonstrated that it is feasible to launch and incorporate an interactive and mentor-based text messaging program within community-based home visitation program settings. Furthermore, our study makes an important contribution in that maternal and child health services, as well as early parenting education programs, usually target mothers (J. Y. Lee et al., 2018). On the contrary, Text4Dad was designed for fathers, especially fathers with low income, and implemented with direct input from such fathers and male CHWs serving those fathers on a daily basis. Another key contribution involves the successful recruitment of fathers, with generally high numbers of fathers enrolled in Text4Dad across the three sites.

Our study had several notable findings. We used an innovative rapid prototyping procedure to develop the text messaging content. Specifically, we recruited fathers from MTurk to provide feedback on the acceptability and relevance of Text4Dad messages. Fathers recruited for all three online surveys had positive reviews of the Text4Dad content. To examine Text4Dad implementation in community settings, separate qualitative interviews with Text4Dad enrolled fathers and all three F-CHWs yielded results that support these findings (J. Y. Lee & S. J. Lee, 2023). Much like the online fathers, fathers enrolled in Text4Dad and the F-CHWs alike reported satisfaction with the Text4Dad content, noting that the text messages were acceptable and relevant to fathers’ parenting experiences. Additionally, the F-CHWs found the technical aspects (e.g., sending individual and group text messages to fathers, technical assistance from the research team) of Text4Dad straightforward and easy to navigate, reporting high levels of satisfaction with the support and the quick resolution of problems (J. Y. Lee & S. J. Lee, 2023). Collectively, these likely aided and resulted in F-CHWs sending out consistent weekly messages to enrolled fathers, as evidenced by the high total numbers of exchanges initiated by the F-CHWs across the three sites.

Another finding to note concerns F-CHWs’ use of different Text4Dad features. Despite efforts to develop acceptable and relevant content across a range of topics during the rapid prototyping process, it was evident that all three F-CHWs were not making use of the curated list of online resources that were embedded within the Text4Dad platform. With the many features available on the platform (e.g., group messages, alerts and reminders, scheduling messages, tailoring messages), it could well be that the F-CHWs overlooked or forgot about the online resource links that were available to them. Similarly, only the F-CHW from Site 3 regularly used the check-in messages built into the Text4Dad platform to elicit responses from fathers. Thus, a recommendation for improving Text4Dad is for the technical assistance team to send out weekly reminders to F-CHWs to share with enrolled fathers, along with example messages with online resource links and check-in prompts embedded in them. This way, the burden to remember the many features of Text4Dad is shifted from the F-CHWs to the technical assistance team.

A key finding from the content analysis is that F-CHWs used Text4Dad mainly to push out information to fathers, especially related to home visit scheduling and local events. That is, the F-CHWs used Text4Dad to communicate with fathers in a unidirectional way, with the goal of information sharing and engaging fathers in in-person fatherhood program activities. Qualitative accounts support these findings as well (J. Y. Lee & S. J. Lee, 2023), with F-CHWs noting that while the development content was useful and informative, they had a low response rate when texting fathers via Text4Dad. The F-CHWs at Sites 1 and 2 saw the main purpose of Text4Dad to be for communicating parenting information and not necessarily engaging in interactions with the enrolled fathers (J. Y. Lee & S. J. Lee, 2023). This is speculative, but discussions with F-CHWs seemed to suggest that they had low expectations for texting as a form of engagement and were perhaps less motivated to use texting to supplement home visits as a result. Indeed, we see that the F-CHWs enrolled many more fathers than we anticipated they would (our initial goal was a total of 30 per site, which was exceeded); however, they did not use the tool to deepen relationships with fathers as we had hoped, but rather as a way to reach more fathers for informational purposes. This is discussed in more detail below. That said, the F-CHWs appreciated the information-sharing aspect of Text4Dad, and they uniformly felt it added value to their fatherhood programming.

Looking at Study 1 and Study 2 together, there is an interesting disconnect between the promise provided by Study 1 (i.e., promising strategies to understand the acceptability and usability of the Text4Dad content) and Study 2 (i.e., strategies that were challenging to implement across the three home visitation sites). Again, two of the three Text4Dad sites did not use Text4Dad as originally intended (e.g., offering support to fathers through bidirectional F-CHW and father interactions). The disconnect between Study 1 and Study 2 is characteristic of what often occurs when implementing program strategies in real-world contexts, where issues of personnel, training, and technology can hinder the application of pre-planned protocols and strategies. Future work should use these critical lessons learned on the ground to modify Text4Dad so that expectations for the program and its implementation in community-based settings are matched and consistent with one another.

### Comparisons between Text4Dad and NurturePA

As noted before, Text4Dad was developed utilizing the mobile technology platform of Nurture PA, which focused on training female volunteers to support postpartum mothers through text messages on child care and development, emotional support, and local parenting resources (Martin et al., 2018; Weiland et al., 2023). Research on NurturePA demonstrated that mothers participated in NurturePA for an average of 296 days in baby’s first year, and actively engaged in bidirectional exchanges with female volunteers related to both child- and mother-focused topics (Martin et al., 2018; Weiland et al., 2023). While both programs utilized a texting platform, NurturePA served a different purpose and intended client population. Mothers were recruited via hospital staff at the time of their children’s births, and the presence of the medical establishment may have lent credibility to NurturePA. Furthermore, the staff of NurturePA devoted a significant amount of time to train and assist the volunteers (for details, see Martin et al., 2018). Compared to NurturePA, Text4Dad only involved a one-hour in-person introduction session and a two-hour virtual training, which entailed going over father-inclusive mentoring principles and the Text4Dad manual, along with demonstration of the software platform. F-CHWs had access to “how-to” videos on the platform, could reach out to the technical assistance team for additional support such as booster sessions, and participated in monthly community learning meetings to exchange helpful information and tips for father engagement in Text4Dad.

The demographic characteristics of the participants in the two programs were also different. NurturePA was implemented as a universal program, and the users were largely white and middle-income women. In contrast, Text4Dad participants were male partners of Medicaid-eligible women from low-income contexts. Contextual factors should not be overlooked. It is worth noting that the participants in Healthy Start face high levels of socioeconomic disadvantage which may have served as a barrier to program participation (S. J. Lee et al., 2011; J. Y. Lee et al., 2018). It may be that the challenge of engaging men with low income is a factor explaining some of the differences in the levels of mentor-mentee interactions.

A key lesson is that the Text4Dad research team may need to implement even stronger training and support for the F-CHWs to facilitate high levels of engagement with fathers, or, alternatively, engage another individual with more dedicated time to send the Text4Dad messages. F-CHWs may have felt that Text4Dad was outside the scope of home visiting services, rather than a central way to increase fathers’ engagement with home visiting. Indeed, paradoxically, Text4Dad was embedded within an existing home visiting program, where fathers were meeting with their F-CHWs fairly regularly, which may have undermined the use of Text4Dad as an engagement tool. Qualitative accounts showed that two of the three F-CHWs felt that in-person home visits, and not Text4Dad, were more appropriate for their interactions with fathers. For example, one F-CHW noted that he liked sending out the messages for informational purposes but specifically preferred to leave interaction to the home visit context (J. Y. Lee & S. J. Lee, 2023). It could be that the demands of the F-CHWs’ full-time jobs as home visitors made it more challenging for them to use Text4Dad. When contrasted with the NurturingPA program, in which there was no home visiting component, mentors had no alternative interaction options outside of texting and their main responsibility was to engage in the texting program. It seems plausible that the NurturePA mentors were better trained and more motivated to fully utilize the texting format as a result, whereas the Healthy Start F-CHWs were expected to rely on home visits for interaction. Additionally, the F-CHWs had relatively low expectations for the use of texting, noting the generally low texting response rate of their fatherhood clients because of their busy schedules and other factors (J. Y. Lee & S. J. Lee, 2023).

### Program and Study Limitations

Importantly, there were lower-than-expected levels of interactivity between the F-CHWs and fathers that were captured on the Text4Dad platform. A number of structural factors may be related to this finding. Again, because Site 2 implemented Text4Dad later than the other two sites, only 48 days had elapsed between when Site 2 started enrolling fathers in Text4Dad and data extraction for analysis purposes was completed. Further, as noted before, Site 1 had an interruption in programming with the F-CHW going on paternity leave, and this may have factored into the F-CHW’s ability to fully utilize Text4Dad, including engaging in ongoing interactions with fathers using the platform. Both of these instances point to an underlying issue regarding programmatic capacity. Healthy Start Engaged Father is a small community-based program, with each site having only one full-time F-CHW to deliver services to fathers. It may be difficult to implement additional programming, such as Text4Dad, in organizational contexts with limited resources. Such programming is likely to be highly impacted by organizational factors, including shortages of staffing, office space, time, and funding.

Another limitation of Text4Dad is that interactions can be inconvenient because the F-CHW had to log in to the Text4Dad platform on a website to respond to fathers’ messages, and one F-CHW indicated as such, noting that the platform which contained the pre-populated evidence-based developmental messages was not particularly user friendly for frequent texting (J. Y. Lee & S. J. Lee, 2023). Although this can be accomplished on a mobile browser, it would still require use of a platform that is not mobile-optimized. A recommendation would be to create a Text4Dad mobile app to aid F-CHWs in more easily sending out and responding to messages from fathers.

Separately, study limitations include not being able to make causal claims as the analyses were primarily descriptive. Furthermore, we have limited data on the sociodemographic characteristics of enrolled fathers (e.g., race) and their infants (e.g., age) because of the inability to build in such fields and/or extract such data on the Text4Dad platform. For instance, being able to examine whether infants’ ages were responsible for the variation in F-CHW-initiated topics of exchanges would have allowed for a more nuanced understanding of factors contributing to differences across the three Text4Dad sites. Comparing program engagement levels and challenges by fathers’ race would have allowed for delving deeper into the data and yielding a more comprehensive understanding of Text4Dad’s effectiveness. It would be critical for future research in this area to collect fathers’ race information and conduct additional analyses by race (e.g., race comparisons in program engagement levels, challenges faced, and the effectiveness of Text4Dad). A related recommendation for future research is to analyze how fathers of different races interacted with Text4Dad and their unique experiences.

Due to the lack of appropriate fields in the Text4Dad platform, we were unable to collect data on in-person activities and home visit sessions fathers attended, which would have yielded key insights in terms of how Text4Dad complemented the in-person home visiting component of Healthy Start, as well as yield information on program dosage and overall father engagement in the program. Future research could consider leveraging existing data, such as linking Text4Dad data with F-CHWs’ caseload administrative data to obtain relevant information. Finally, our study results cannot be generalized to other fathers from low-income contexts.

## Conclusion

As of April 2019, the Health Resources and Services Administration (HRSA) has stipulated that Healthy Start programs ought to aim to involve 100 fathers or father surrogates per year in services and activities. Thus, Healthy Start programs may be motivated to use a supportive program, such as Text4Dad, to meet HRSA’s goal. The Text4Dad research team is actively working with the Healthy Start program sites to bolster program implementation and use. However, additional efforts are needed to establish that Text4Dad indeed increases fathers’ home visitation attendance and engagement, and can be useful for Healthy Start in engaging harder-to-serve fathers. Leveraging mobile technology and specifically text messaging, the current study demonstrated the promise of a low-cost, mentor-based add-on for supporting and engaging fathers in the context of home visits, and serves as a potential model of how to systematically develop similar interventions.

## Data Availability

Please reach out to Joyce Y. Lee (lee.10148@osu.edu) for data or research project materials.

## Code Availability

Please reach out to Joyce Y. Lee (lee.10148@osu.edu) for code.
